# Surface Coating of Polyurethane Films with Gelatin, Aspirin and Heparin to Increase the Hemocompatibility of Artificial Vascular Grafts

**DOI:** 10.34172/apb.2023.013

**Published:** 2022-01-03

**Authors:** Simzar Hosseinzadeh, Forough Shams, Roya Fattahi, Ghader Nuoroozi, Elnaz rostami, lida Shahghasempour, Nasim Salehi-Nik, Mahboubeh Bohlouli, Arash Khojasteh, Nazanin Ghasemi, Habibollah Peiravi

**Affiliations:** ^1^Medical Nanotechnology and Tissue Engineering Research Center, Shahid Beheshti University of Medical Sciences, Tehran, Iran.; ^2^Department of Tissue Engineering and Applied Cell Sciences, School of Advanced Technologies in Medicine, Shahid Beheshti University of Medical Sciences, Tehran, Iran.; ^3^Department of Medical Biotechnology, School of Advanced Technologies in Medicine, Shahid Beheshti University of Medical Sciences, Tehran, Iran.; ^4^Department of Animal Sciences and Biotechnology, Faculty of Life Sciences and Biotechnology, Shahid Beheshti University, Tehran, Iran.; ^5^Department of Microbiology, Karaj Branch, Islamic Azad University, Karaj, Iran.; ^6^Department of Immunology, School of Medicine, Shahid Beheshti University of Medical Sciences, Tehran, Iran.

**Keywords:** Hemocompatibility, Polyurethane, Heparin, Aspirin, Surface modification

## Abstract

***Purpose: *** A hemocompatible substrate can offer a wonderful facility for nitric oxide (NO) production by vascular endothelial cells in reaction to the inflammation following injuries. NO inhibits platelet aggregation this is especially critical in small-diameter vessels.

***Methods:*** The substrate films were made of polyurethane (PU) in a casting process and after plasma treatments, their surface was chemically decorated with polyethylene glycol (PEG) 2000, gelatin, gelatin-aspirin, gelatin-heparin and gelatin-aspirin-heparin. The concentrations of these ingredients were optimized in order to achieve the biocompatible values and the resulting modifications were characterized by water contact angle and Fourier transform infra-red (FTIR) assays. The values of NO production and platelet adhesion were then examined.

***Results:*** The water contact angle of the modified surface was reduced to 26±4^∘^ and the newly developed hydrophilic chemical groups were confirmed by FTIR. The respective concentrations of 0.05 mg/ml and 100 mg/mL were found to be the IC50 values for aspirin and heparin. However, after the surface modification with aspirin, the bioactivity of the substrate increased in compared to the other experimental groups. In addition, there was a synergistic effect between these reagents for NO synthesis. While, heparin inhibited platelet adhesion more than aspirin.

***Conclusion:*** Because of the highly hydrophilic nature of heparin, this reagent was hydrolyzed faster than aspirin and therefore its influence on platelet aggregation and cell growth was greater. Taken together, the results give the biocompatible concentrations of both biomolecules that are required for endothelial cell proliferation, NO synthesis and platelet adhesion.

## Introduction

 Cardiovascular disease is one of the leading causes of death worldwide. Currently, autologous grafts are the best available clinical practice for vascular grafts or bypass surgeries. However, there are some restrictions on the use of autologous grafts in patients with vascular disease, such as the need for an additional surgery and the lack of vascular sources.^1^ The inner vascular layer is lined with endothelial cells to prevent clot formation. The delivery of antiplatelet or anticoagulant agents such as thrombomodulin,^2^ albumin^3^ and heparin^4,5^ is an emergency to reduce clot formation in artificial vessels. Therefore, a special graft with an optimized surface is necessary for the adhesion and growth of vascular endothelial cells. Several studies have focused on improving the function of artificial vessels through the use of different surface modification strategies. In these studies, mainly polytetrafluoroethylene (ePTFE) or polyethylene terephthalate (Dacron) were used to fabricate artificial vessels with large or medium diameters.^6^ However, after a while due to thrombosis and immune response, they were useless.^7^ Polyamides such as polyurethane (PU) with sufficient elasticity properties and high resistance to mechanical forces such as pulse pressures from blood flow, were presented as good candidates for the establishment of artificial vessels. While, this synthetic polymer has a limitation in cellular connections.^4^ On the other hand, collagen, gelatin and fibronectin are abundant proteins in the extracellular matrix (ECM), which lead to better endothelial cell adhesion.^8^ In this regard, the stable coating of biomaterials with ECM proteins, can increase cellular adhesion and growth. In addition, these proteins stimulate platelet adhesion, but once they become detached from the surface, cellular separation occurs under shear stress. Then, this event leads into clot formation and vessel obstruction.^4^ It can be concluded that a suitable coating for endothelial cell adhesion and better blood compatibility are achieved, if the coating components contain anticoagulants. Among much researches on surface modifications, plasma treatment is well-known as a reproducible technique to reduce the surface hydrophobicity of synthetic vessels containing ePTFE and Dacron.^8^ Therefore, this method increases the interactions between the substrate surface and endothelial cells and consequently improves cell adhesion and proliferation without stimulating platelet adhesion.^9^ In another study by Dimitrievska et al, polyethylene glycol (PEG) was used to improve the blood tolerance of commercial Dacron and the related results concluded that the 10% PEG solution showed the best effect.^10^ Moreover, Hashi et al employed PEG as a linker between hirudin polypeptides and poly-L-lactide acid (PLLA) scaffold. The results confirmed that, the implant in the carotid artery had the vessel patency of 75% after 1 month compared to 50% in the unmodified surface. Therefore, the surface modification could significantly reduce platelet coagulation.^11^

 In this study, the PU films were produced in a casting process and chemically modified by plasma treatments. These surfaces were examined for their chemical changes using the related methods. The substrates were immobilized by the following molecules and their effects on cell attachment and proliferation was studied. In addition, platelet aggregation and nitric oxide (NO) production were examined to reveal the differences between the scaffold groups. PEG and gelatin were chosen as coating agents in order to increase the cell activity of the substrates. Human umbilical vein endothelial cells (HUVEC) cell line was the cell source in this investigation. Also, aspirin and heparin were cross-linked on the surface of the scaffolds by chemical reactions to apply their anti-coagulation activity. The most important goal of this study was to establish a surface with the lowest coagulation potency and the highest cell adhesion. For this, the optimum concentrations of these biomolecules including aspirin and heparin were obtained. Moreover, the associated events of platelet adhesion and NO synthesis were searched and compared between the various test and control groups. Overall, at the end of this study, a substrate with special chemical and biological properties was recommended for the manufacture of artificial vessels especially for the types with small diameter.

## Materials and Methods

 Polymers including thermoplastic PU and PEG (2000 MW) were obtained from Desmopan (Leverkusen, Germany) and Sigma-Aldrich (Shanghai, China) respectively. However, gelatin was purchased from Sigma-Aldrich (St. Louis, MO, USA). The solvents of tetrahydrofuran (THF), ethanol, glutaraldehyde, dimethyl sulfoxide (DMSO), hexane, hydrochloric acid (HCL) and the cross-linkers of 1-Ethyl-3-(3-dimethylaminopropyl) carbodiimide (EDC)/N-Hydroxysuccinimide (NHS) were bought from Merck (Hohenbrunn, Germany). The other chemical materials such as sulfanilamide, naphtylethelenediamine-dihydrochloride, NaNO_2_ and H_3_PO_4_ were also purchased from Merck too. Aspirin, toluidine blue, CaCl_2_ and 3-[4,5-dimethylthiazol-2-yl]-2, 5-diphenyl tetrazolium bromide (MTT) were provided from Sigma-Aldrich (St. Louis, MO, USA). Heparin (Heparin sodium injection parenteral, 5000 U/mL) was provided by DarouPhakhsh (Tehran, Iran). The cell culture medium Dulbecco’s modified Eagle’s medium (DMEM) with high glucose content, fetal bovine serum (FBS), trypsin and phosphate buffered saline (PBS) were purchased from Gibco (Grand Island, NY, USA). Moreover, the HUVEC line was purchased from the Pasture Institute cell bank (Tehran, Iran).

###  Production of PU film and modification of its surface by PEG, gelatin, heparin and aspirin 

 For the production of PU films, the solvent casting method was used to evaporate the solvent. This method was carried out by preparing a solution of PU with a concentration of 6% in THF. This solution was poured into a 48-well plate after dissolving the polymers. The plate was placed on a hot plate at 60°C for 2 hours and the resulting membranes were several times washed with PBS and completely dried with a freeze drying apparatus at -40°C. For surface modification, a plasma generator (Diener Electronics, Germany) with a quartz reactor was used at a frequency of 44 GHz in an ambient atmosphere for 20 min. The treated films were immersed in a solution of EDC and NHS as chemical coupling agents at a concentration of 5 mg/mL for 12 hours. The PEG and gelatin solutions were prepared in the respective concentrations of 0.02 g/mL in ethanol and 0.001 g/mL in distilled water and poured into the wells. Moreover, the membranes were treated with aspirin and heparin in the optimal concentrations obtained. The experimental groups were the films which grafted with PEG, gelatin, gelatin-aspirin, gelatin-aspirin-heparin and gelatin-heparin as well as the plasma treated PU and TCPS groups. The PEG group was regarded as a negative control, TCPS as a positive control and the other groups as test groups. Before the cell culture process, scaffold samples were sterilized by UV irritation for 20 min and incubation in 70% ethanol for 40 minutes. In addition, glutaraldehyde (4%) was used for cell fixation and the serial ethanol concentrations of 50% to 100% for cell dehydration in SEM studies.

###  Optimal concentrations of aspirin and heparin

 The anticoagulant effects of factors such as aspirin and heparin should be investigated in biocompatible and effective concentrations. For this approach, a concentration gradient of these agents was applied on the HUVEC cell line and cell viability was assessed by MTT scoring. The concentrations were 0.01, 0.03, 0.05, 0.08, 0.1 and 0.15 mg/mL for aspirin and 1, 10, 15, 30, 60 and 100 mg/mL for heparin and the corresponding tests were carried out after 24, 48 and 72 hours. At those times, the cells were washed with PBS and treated with 0.1% MTT solution in high glucose DMEM without FBS. The cells were incubated for 3.5 hours in a 5% CO2 incubator and 37°C under dark conditions. Then, DMSO was added to solubilize the formazan crystals and their absorbance values at 570 nm were read using a UV spectrophotometer (Pharmacia Biotech, Germany). The relative growth rate (RGR) was calculated using the following formula:


$$RGR\%=\frac{OD\;t}{OD\;c}\times 100$$

Where, ODt is the abbreviation for optical density and defines the light absorbance of the test groups and ODc relates to the control group.

 In addition, for evaluating scaffold cell viability, the exact same protocol for measuring cell viability by MTT assay was repeated. However, when DMSO was added to dissolve the formazan crystals, the scaffolds were vortexed for 30 seconds in the presence of glass beads to release any formazan molecules.

###  NO measurements of the treated HUVEC cell line 

 Nitrite measurements were carried out with 0.5 mL of the cell medium. Then, the Griess reagent contained 1% sulfanilamide, 0.1% naphtylethelenediamine-dihydrochloride and 2.5 M H_3_PO_4_ was added to this cell medium. Additionally, a dilution of the NaNO_2_ series in DMEM was prepared at a concentration of 1, 3, 6, 12, 25, 50 µg/mL. The curve obtained was used as a standard curve in order to convert OD values into concentrations. The medium samples were then mixed with the Griess solution and their absorbance value at 545 nm was obtained using a UV spectrophotometer. The assay was repeated when cells were seeded on PU membranes grafted with PEG, gelatin, gelatin-heparin, gelatin-aspirin and gelatin-heparin-aspirin. The test was done for TCPS as a control group.

###  Examination of platelet adhesion on PU films

 The hemocompatibility of the experimental groups, which included heparin, aspirin and heparin-aspirin, was investigated using the platelet adhesion method. Briefly, a fresh platelet rich plasma (PRP) was obtained from a healthy volunteer. A CaCl_2_ solution (20 mM) for this evaluation was added to the PRP solution and physiological saline solution at a ratio of 1:1. The mixture then was stored at 37°C for 1 hour. The developed gel was mechanically dissected and finally, the sample was centrifuged for 10 minutes at 3000 rpm. The upper part of the solution was aspirated and incubated for 48 hours at 4°C. After this time, the solution was centrifuged again and the supernatant was filtered for its sterilization. It should be noted that the freeze-thaw method was used for 3 times between -22 and 37°C to activate the PRP solution. After the activation, the corresponding solution was centrifuged again, but at 4°C.^12^ The PU films including grafted PEG, gelatin, gelatin-heparin, gelatin-aspirin and gelatin-heparin-aspirin were examined for the their hemocompatibility after sterilization. Therefore, about 200 μL PRP was poured into the films and incubated for 1 hour at 37°C. The wells were then washed for several times using PBS to eliminate the platelets that adhere weekly to the scaffolds.^13^ The platelet adhesion number was obtained by using a hematology analyzer (KX-21 N, Sysmex, Norderstedt, Germany). The percentage of platelet adhesion was calculated using the following formula^14^:


$$Platelet\;adhesion\;\%=\frac{Ps}{Pc}\times 100$$

Where Ps is the number of platelet on the scaffolds after their incubation and Pc is the PRP value of the PEG-modified PU film as the negative control since PEG could inhibit platelet adhesion according to previous studies.^15^

###  Stability of heparin and aspirin surface modification on PU films 

 With regard to the stability of the surface modification by aspirin and heparin, the release of these reagents was studied for 10 days. For the individual studies on aspirin and heparin release, the PU films which were immobilized by aspirin or heparin, were immersed in PBS and placed in a 37°C incubator. Their supernatants were collected after 1, 2, 3, 4, 5, 6, 7, 8, 9 and 10 days and replaced with PBS. The amount of aspirin was characterized by measuring the absorbance of the sample at 305 nm. However, the absorbance values were changed to concentrations (%) using a standard curve.^16^ On the other hand, the colorimetric method with toluidine blue was adopted to obtain the released heparin. The toluidine blue dye (0.005%) was prepared by dissolving in HCL (0.01 N). Approximately, 200 µL of the supernatant was added to 2.5 mL of the dye. The samples were shaken for 1 minute and then, hexane (5 mL) was poured in to form a heparin dye-containing layer. The layer was used to detect the heparin concentrations at 631 nm. The process was repeated after the aspirin-like time intervals. An associated standard curve was used to report the heparin concentrations.

###  Contact angle measurements and FTIR spectroscopy of the pristine and plasma-treated PU films 

 The surface wettability of the PU films including the plasma-treated and untreated, was studied by examining the contact angle with water. The test was performed using a G10 Kruss contact angle goniometer after the scaffold samples were assembled on their respective stages. The angle was determined with the software ImageJ (version 2015) using a sessile drop technique at 25°C after 10 s. Also, the PU scaffolds including plasma treated and untreated types, were evaluated by means of Fourier transform infra-red (FTIR) spectroscopy (Thermo Nicolet model: NEXUS 670, USA). The spectrum was recorded against a background of KBr pellet with a wavelength range of 4000 - 500 cm ^-1^. Each FTIR spectrum was obtained with the resolutions of 2 cm ^-1^.

###  Statistical analysis

 Values with significant differences between the groups were evaluated with the Sigma Plot (version 2017). The *t* test and Mann-Whitney methods were chosen to show the significant difference between 2 or more than 2 groups, respectively. If *P *values were less than or equal 0.05, the difference was reported as significant and for the higher values, the difference was indistinguishable. All data were discussed as mean ± SD that the mean was the average of at least 3 data in a test and the SD as the representative of standard deviation. Additionally, the normality of the data was important in order to initiate a statistical analysis.

## Results and Discussion

###  Investigation of HUVEC proliferation by MTT assay after heparin and aspirin treatments

**Figure 1:**
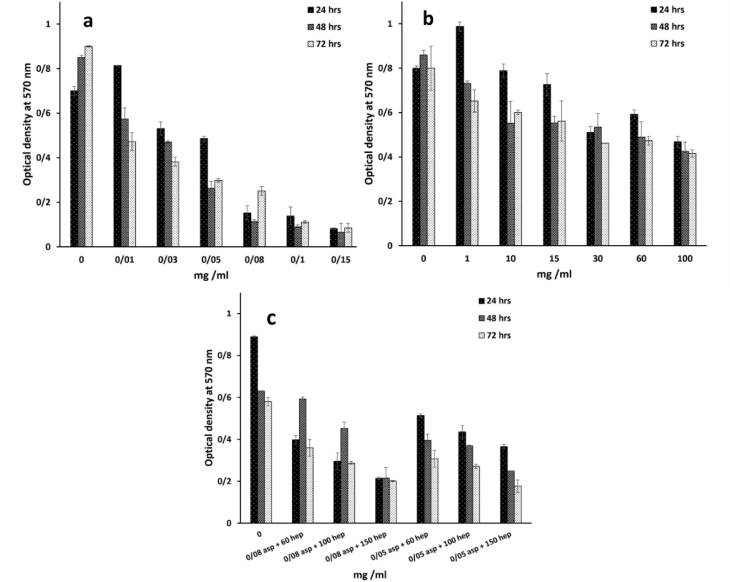
HUVEC cells were treated with a gradient concentration of aspirin (a), heparin (b) and both aspirin + heparin (c) to find out their IC50 points by MTT assay. The analysis was done after 24, 48 and 72 hours. Asp and hep are the abbreviations of aspirin and heparin respectively

**Figure 2:**
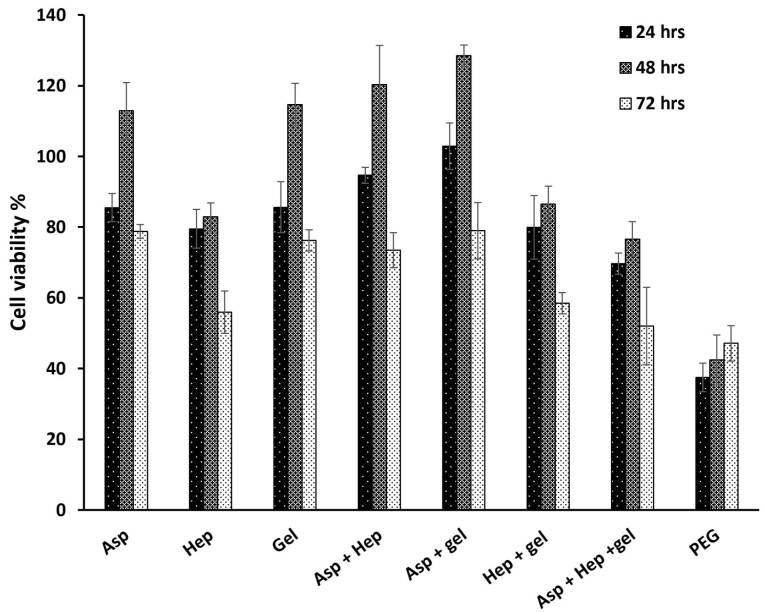
HUVEC cell viability after their culture on the PU films coated by aspirin, heparin, gelatin, aspirin + heparin, aspirin + gelatin, heparin + gelatin, aspirin + heparin + gelatin and PEG. Herein, PEG was considered as the negative control. The analysis was done after 24, 48 and 72 h and the respective concentrations of aspirin and heparin were 0.05 and 100 mg/mL. Asp, hep and gel are the abbreviations of aspirin, heparin and gelatin respectively. The OD values of the experimental groups were normalized by TCPS as the non-treated group

 Optimal concentrations of heparin and aspirin were required to assess their anticoagulation and NO production respectively. According to previous reports, the higher aspirin concentration of 4 mmol/L could cause acute toxicity in patients.^17^ This is in line with our findings that the high concentrations of 0.05, 0.03 and 0.01 mg/mL (Figure 1a) at the respective time points of 24, 48 and 72 hours, reduced the percentage of cell viability to less than 50% (Table 1). The effective dose of aspirin, which has been used for the acute treatment of coronary disorders, is 75-325 mg/day.^18^ If the maximum biocompatible amount is compared with this value, the corresponding dose is close to our value despite a total blood volume of approximately 5 L. As can be seen from Table 1, the toxicity of the treatment dose of 0.08 mg/mL, reduced the percentage of cell viability by 19.35%, 15.05% and 30.31% after 24, 48 and 72 hours, respectively. On the other hand, the toxic value of heparin was determined after 100 mg/mL (Figure 1b and Table 2) and the cell viability of the corresponding dose was reduced to half the value of the control group (IC_50_ point, *P*> 0.05). Heparin as an anticoagulant component, could inhibit endothelial cell proliferation,^19^ but it is able to increase cell proliferation in the presence of basic fibroblast growth factor as a cofactor.^20^ Herein, in the absence of growth factors but in the presence of FBS, it has been predicted that cell viability is reduced. In accordance with the data, heparin had a critical concentration to inhibit cell proliferation. A previous study confirmed that a heparin concentration of 1 µg/mL, could reduce cell proliferation to 70%.^21^ In contrast, this study showed that there was no inhibition of cell growth at the concentrations of ≤ 1 mg/mL. But the highest concentration of 100 mg/mL decreased cell viability to 53.62%, 49.53% and 52.12% at the time points of 24, 48 and 72 hours. Another study reported that the IC50 points at the same time points as the present study, were 0.6, 0.5 and 0.4 mg/mL.^22^ These different values between the reports could be due to the differences in cell type and culture conditions. In this regard, if heparin level exceeds from the critical concentration, it could be toxic. In particular, it has been approved that heparin can decrease NO production by endothelial cells in high concentrations. The results of the aspirin and heparin combination were then re-examined to find out a combined concentration of both reagents that can inhibit cell viability by half compared to TCPS (Figure 1c). The aspirin and heparin combination values were 0.08 mg/mL at 60 mg/mL, 0.08 mg/mL at 100 mg/mL, 0.08 mg/mL at 150 mg/mL, 0.05 mg/mL at 60 mg/mL, 0.05 mg/mL at 100 mg/mL and 0.05 mg/mL at 150 mg/mL. Under these treatment conditions, 0.05 mg/mL aspirin and 100 mg/mL heparin decreased cell viability to 51.29%, 61.54% and 68.59% after 24, 48 and 72 hours. Here, this concentration was considered to be the specific concentration of aspirin and heparin that could not inhibit cell viability by more than 50%. So, this point was used to modify the surface of PU films to obtain a blood compatible membrane for vascular tissue engineering. The corresponding factors were grafted through covalent bonds onto the surface of PU films after their treatments with plasma.^23^ Here, the PU modified with PEG 2000 was used as a negative control and its lower cellular activity represented the values of 37.51%, 42.44% and 47.15% for the time points of 24, 48 and 72 hours, respectively (Figure 2).^24^ In contrast to this, the PU-gelatin mat had the significantly highest cell viability of 85.60%, 114.06% and 76.26% for the respective times compared to the PU-PEG group (*P* < 0.05). As discussed above, the gelatin surface coating improves not only endothelial cell proliferation, but also their spreading due to the presence of arginine-glycine-aspartic acid (RGD) peptides with this polymer for cell attachment.^25^ The hypothesis about the higher hemocompatibility, which was developed after the application of aspirin and heparin in the PU-gelatin films, could be well understood when considering the other groups. The bioactivity of PU films improved after their grafting with gelatin and aspirin molecules for HUVEC cells especially in the absence of heparin. When the substrate was modified with aspirin, after the gelatin coating, the cellular bioactivity values were 102.87%, 128.52% and 79.03%. While, the cell proliferation in this group in the presence of heparin was reduced to 94.64%, 120.32% and 73.46%. However, the difference was not significant statistically (*P* > 0.05). The result was consistent with an earlier report that confirmed that the heparin coating cannot affect cell proliferation.^26^ In our study, the presence of heparin attenuated the bioactive property of aspirin. In this way, after the PU film was coated only with heparin, the cell viability reached to the lowest level representing the anti-proliferative activity of heparin as discussed above. The cell viability values for this group were 79.51%, 82.89% and 55.95% in the time intervals of 24, 48 and 72 hours after cell culture. The result explains the depressant effect of heparin on the growth of HUVEC cells.

**Table 1:** HUVEC cell viability after their treatments with aspirin at a gradient concentration between 0.01 - 0.15 mg/mL

**Concentrations aspirin / time points**	**0.01** **mg/mL**	**0.03** **mg/mL**	**0.05** **mg/mL**	**0.08** **mg/mL**	**0.1** **mg/mL**	**0.15 mg/mL**
24 h	103/92	76/28	49/5	19/35	18/5	11/14
48 h	67/05	51/17	32/05	15/05	10/82	7/94
72 h	50/66	43/72	35/05	30/31	12/78	9/47

The OD values of the experimental groups were normalized by TCPS as the non-treated group.

**Table 2:** HUVEC cell viability after their treatments with heparin at a gradient concentration between 1 - 100 mg/mL

**Concentrations heparin / time points**	**1** **mg/mL**	**10** **mg/mL**	**15** **mg/mL**	**30** **mg/mL**	**60** **mg/mL**	**100 mg/mL**
24 h	113/25	94/46	88/62	75/71	80/51	53/62
48 h	85/11	64/06	64/30	62/20	56/86	49/53
72 h	81/50	75/12	70/25	57/75	59/12	52/12

The OD values of the experimental groups were normalized by TCPS as the non-treated group.

###  NO production after cell treatments at optimal heparin and aspirin concentrations 

**Figure 3:**
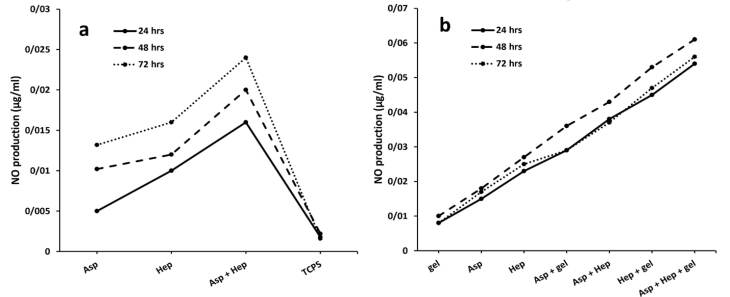
HUVEC cell NO synthesis after their treatments with aspirin, heparin, aspirin + heparin and TCPS as the non-treated group (a) and after their culture on the PU films modified with aspirin, heparin, gelatin, aspirin + heparin, aspirin + gelatin, heparin + gelatin and aspirin + heparin + gelatin. (b) The analysis was done after 24, 48 and 72 h and the respective concentrations of aspirin and heparin were 0.05 and 100 mg/mL. Asp, hep and gel are the abbreviations of aspirin, heparin and gelatin respectively

 A standard curve of NaNO_2_ solutions in a serial dilution should convert the OD values of the NO molecules produced into µg/mL. As in before studies, aspirin stimulates the production of NO by endothelial cells and its concentration of 50 nM is enough to release NO instantly.^27^ The presence of NO causes hemocompatibility^27^ by preventing platelet adhesion and its role as a vasodilator,^28^ although it has a short half-life.^29^ In the present study, the NO synthesis was measured in the absence of the PU films between the treatment groups of aspirin, heparin and aspirin + heparin. In Figure 3a, it is clear that the group treated with both reagents, had the highest level of NO production. This finding was in agreement with previous studies that confirmed that aspirin activates NO synthesis in plasma to reduce local inflammation.^30^ In addition, one study reported that aspirin at a concentration of 3-30 µmol/L protected endothelial cells from hydrogen peroxide toxicity. The corresponding value of 0.05 mg/mL completely agreed with its optimal concentration in the present study. In contrast to the inhibitory effect of heparin at high concentrations on NO production, its optimal exposure potentiates the activity of NO synthase in endothelial cells through a Gi-protein mechanism.^31^ It has also been approved that this factor protects endothelial cells by mitogen-activated protein kinase and NF- κB signaling pathways. Therefore, the synergistic influence of aspirin and heparin on nitric acid synthesis, is completely significant compared to the other groups (Figure 3b). Later, heparin was stronger than aspirin to induce NO synthesis (*P*< 0.05) and it could be concluded that the highest proportion of the NO development in the treated group was related to heparin. Moreover, the NO synthesis was examined in the PU films that were immobilized with aspirin, gelatin, heparin, aspirin + gelatin, aspirin + heparin, gelatin + heparin and aspirin + heparin + gelatin. Here, the lowest value (µg/mL) related to the groups that were only coated with gelatin. It seems that due to the covalent bonds^32^ between the PU films and factors such as gelatin, aspirin and heparin, their release would be impossible. However, slight hydrolysis reactions or even the enzymatic cleavage of the corresponding bonds ensure the diffusion of these active ingredients from the grafted substrates. The indistinguishable relationship between PU-PEG and PU-gelatin, confirmed that there is no significant NO activity after gelatin coating (*P* > 0.05). The groups comprising the PU-gelatin-aspirin, PU-gelatin-heparin and PU-gelatin-aspirin-heparin showed significant differences compared to the PU-PEG group. According to the results of the treatments without the PU films, the group treated with the all compounds, showed a higher value of NO synthesis. When, heparin is grafted onto the PU membranes due to its more carboxylic chemical groups compared to aspirin, it was expected that its release would be slower than aspirin. This phenomenon can lead to lesser influence of heparin on NO synthesis. In contrast, due to the more hydrophilic nature of heparin compared to aspirin, its hydrolysis is faster and results in greater absorption by cells. In summary, it can be said that heparin has more bonds with the substrate surface which can reduce its activity in NO production. While, this biomolecule is further degraded by water due to its hydrophilic property. Thus, heparin could easily have a synergistic relationship with aspirin in terms of NO production. Even, the greater proportion of this phenomenon in the latter group may be related to heparin rather than aspirin. In addition, it has been approved that the higher concentrations of aspirin stop the protein expression of nitric acid synthase.^33^ The aspirin concentration reported in a study was 0.25 mmol/L,^33^ which is very close to the treatment dose in this study (0.05 mg/mL).

###  Platelet adhesion of PU films after gelatin, aspirin and heparin grafting 

**Figure 4:**
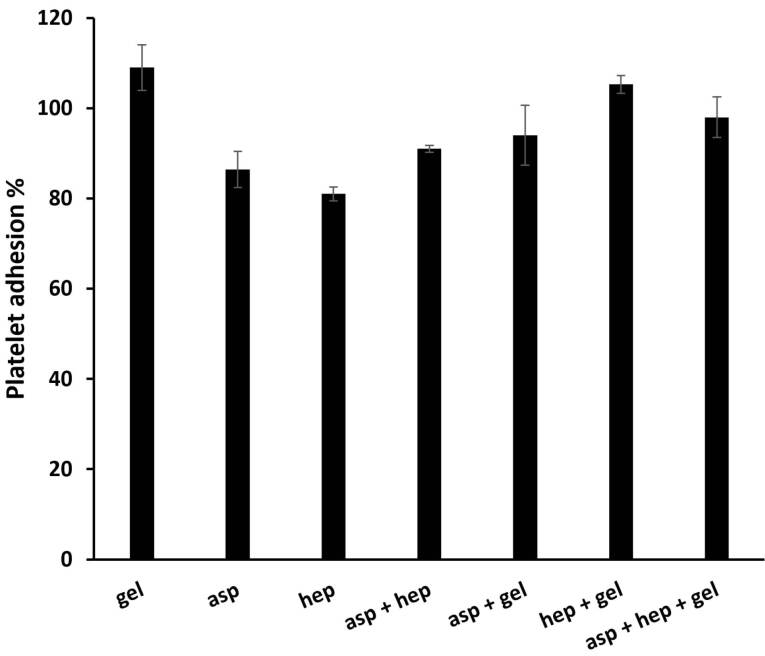
The platelet activity of the PU films coated with aspirin, heparin, gelatin, aspirin + heparin, aspirin + gelatin, heparin + gelatin and aspirin + heparin + gelatin. The analysis was done after the incubation of the samples at 37°C for 1 h. The respective concentrations of aspirin and heparin were 0.05 and 100 mg/mL. Asp, hep and gel are the abbreviations of aspirin, heparin and gelatin respectively. Herein, PEG was employed as the negative control to normalize the experimental groups

 After endothelial dysfunction, platelet adhesion is of crucial importance for the fibrin formation of these cells.^34^ Although, the normal endothelial cell layer inhibits this occurrence by producing NO.^35^ A hemocompatible substrate as a scaffold for endothelial cells, must not only support their growth, but also inhibit platelet aggregation. After plasma treatments, the PU membranes were grafted with PEG, gelatin, gelatin-aspirin, gelatin-heparin and gelatin-aspirin-heparin factors in the presence of chemical cross-linkers (EDC/NHS). To explain the statistical differences between the membrane groups in terms of platelet adhesion, the PEG group was used as a negative control, which does so through its attractions to Ca^2 +^ ions during platelet function.^36^ Additionally, both heparin and aspirin could inhibit this process and consequently the kinetics of whole blood clotting could be reduced. Heparin, a well-known anticoagulant, has been approved as a reducing factor for platelet adhesion^37^ by improving activated partial thromboplastin time.^23^ If heparin is applied to artificial grafts, it would therefore decrease their platelet function.^38^ On the other hand, aspirin also has a strong inhibitory effect on platelet activity in the intact layer of endothelial cells^39^ and motivates the function of NO synthase.^14^ Moreover, aspirin triggers this function regardless of its concentration and at both high and low dosages of exposure, it is capable of inhibiting platelet function.^16^ As is obtained from Figure 4, the PU-gelatin scaffold induced platelet adhesion due to its highest bioactivity. In contrast, the surface chemically modified by aspirin, decreased platelet aggregation to 86.46 ± 4, representing the high activity of aspirin against this incident. Here, the surface containing heparin reduced platelet function which is significantly lower than that of aspirin (*P* < 0.05). In this way, it should be noted, that heparin due to its higher hydrophilic property compared to aspirin, may show a stronger potential result in this phenomenon. However, in the heparin and aspirin combination group, there was no synergistic function between these factors and the value reached to 91.21 ± 0.8%. This finding was confirmed by previous studies that heparin might weaken the antiplatelet activity of aspirin. On the other hand, the addition of gelatin to the groups of aspirin, heparin and their combination group, gave the highest values of platelet adhesion and it is evident for all groups. This observation is due to the high ability of gelatin to adhere to platelets and therefore, can increase blood clotting.

###  Stability of surface modification with aspirin and heparin

**Figure 5:**
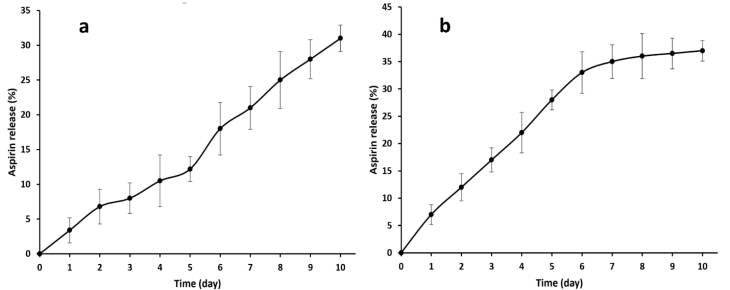
The stability of the surface modifications with aspirin (a) and heparin (b). The analysis was done after the incubation of the samples at 37°C for 1-10 days. The respective concentrations of aspirin and heparin were 0.05 and 100 mg/mL

 The superficial immobilization of aspirin and heparin must be maintained for at least several days, so that it can perform its function as a vascular implant. However, when the coated factors are administered explosively, the toxic results occur with no beneficial effect on NO synthesis and platelet adhesion. The OD values (at 305 nm) of the released aspirin were converted into the concentrations using a standard curve and these dosages are given here in percent after taking into account the first weight of aspirin that was applied to the PU films. As can be seen, the slope of this curve is positive and shows a gradual accumulative pattern for aspirin release (Figure 5a). There is no burst release and the curve shows a controlled rate between 1 and 10 days. However, the levels of the release are highest in the first 2 days, suggesting the presence of free aspirin molecules on the scaffold surface. The release mechanism of aspirin is related to diffusion and PU degradation may not be involved due to its non-biodegradable nature.^40^ In addition, the OD values of released heparin were changed in concentrations and then in percent. In contrast to a simple method of measuring aspirin release, a colorimetric method was used to determine the amount of heparin administered.^41^Figure 5b shows the heparin release profile of the PU film. It should be noted that both patterns resulting from the release of aspirin and heparin obey zero order kinetics at all times due to their stable release dose. However, there are some fluctuations that recommend the first order model to some extent. It appears that there is no initial burst release and the release percentage of heparin has reached 33.14 ± 2% after 6 days. After that, a plateau was developed up to the last day. In this way, its first region has a positive slope like aspirin, which confirms the release of heparin by Fickian diffusion. Although, the late part is related to the destruction of the concentration gradient established in the first stage. In other words, this initial driving force is destroyed in the next after 6 days^42^ and the release value is thus fixed. It is expected that when this scaffold is implanted in the body, due to the breakdown of heparin by cellular enzymes, this plateau will be removed and the diffusion mechanism will survive.

###  Contact angle measurements and FTIR spectroscopy of PU films 

**Figure 6:**
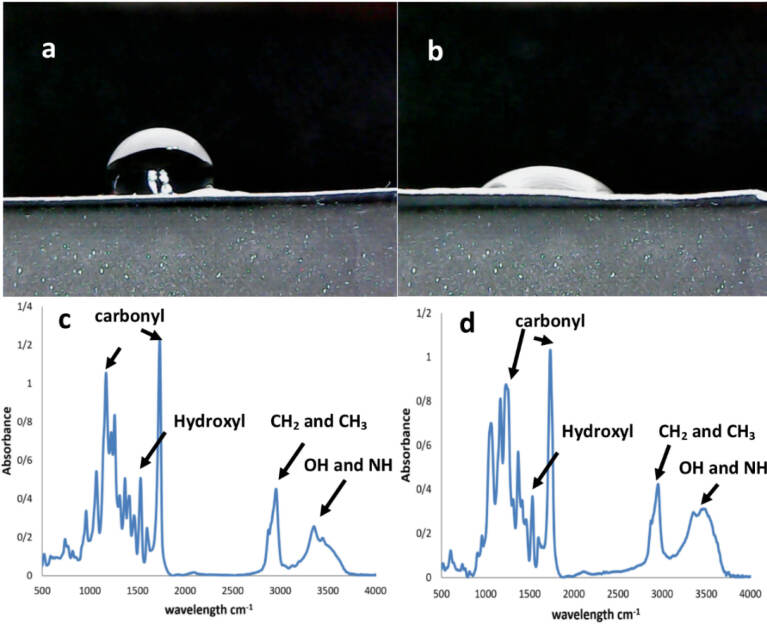
The water contact angle of PU films before plasma treatment (a) and after plasma treatment (b). Also, the FTIR spectra of PU films before plasma treatment (c) and after plasma treatment (d)

 Static contact angle measurements were performed for the PU films including the original and treated types. The angles reported in this study refer to 10^th^ s after a drop of water is applied. It is obvious that the contact angle with water is decreased due to the higher wettability after plasma treatments in atmospheric pressure.^43^ The use of substrates with a hydrophilic surface is essential for proper cell spreading and morphology. These materials should ensure extensive interactions between cell membrane and their surface.^44^ In this document, the corresponding value of 92 ± 6^∘^ was obtained for the untreated PU membrane (Figure 6a). In this regard, a contact angle greater than 80^∘^ cannot provide a biocompatible surface for cells.^45^ Conversely, the treated films gave a smaller water contact angle of 26 ± 4^∘^, which confirms the successful preparation of a highly hydrophilic surface for cell interactions (Figure 6b).^46^ The differences between these scaffold types were statistically significant (*P* < 0.05) and described the remarkable role of plasma treatments in the surface engineering of scaffolds. Next, FTIR spectroscopy was used to demonstrate the formation of functional groups after the plasma processing. The method was able to investigate the chemical nature of the materials used on the basis of the vibration, stretching, bending and rotation mutations of surface molecules. Here, the treated PU membrane was compared with the pristine PU film in the length range of 4000 up to 500 cm ^-1^. As can be clearly seem Figure 6c-d, the untreated PU scaffold had a specific broad peak at 3500 cm ^-1^ which is relevant for the N-H stretching.^47^ There was also another sharp peak from the symmetric oscillation of CH_2_^48^ and CH_3_^49^ at 2900-2800 cm ^-1^. Later, another small peak appeared at 1530 cm ^-1^, which could be related to O-H deformations.^50^ In contrast, a large peak between 1750 and 1800 cm ^-1^ is attributed to the stretching vibration of carbonyl groups according to previous studies.^51^ Some complicated absorption bands between 1100 and 1200 cm ^-1^ were found for this polymer, which are representative of non-symmetrical C–O–C and aromatic C–O stretching vibrations, respectively.^52^ Moreover, a sharp peak associated with the carbonyl groups could be seen at 1726 cm ^-1^ ^53^ with both scaffold groups. However, after plasma treatments to convert the surface of a hydrophobic substrate into a wettable one, the peaks of the original PU mat were partially changed. The intensity of these specific peaks associated with the asymmetrical stretching mode of CH_2_ and CH_3_ at 2910 and 2955 cm ^-1^, was increased.^54^ In addition, the peak of 3500 cm ^-1^ which could be assigned to the newly developed O-H groups, had been improved.

## Conclusion

 For adequate hemocompatibility in artificial vessels, a scaffold with lower platelet function is required. But, this lower capacity must have sufficient hydrophilicity or even cell-binding ligands to provide a surface for the adhesion of endothelial cells. On the other hand, this substrate must drive these cells to synthesize NO. In this case, a substrate with the improved hemocompatibility was recommended for therapeutic approaches in vascular tissue engineering. It appears that a PU film could be an acceptable candidate as a substrate due to its mechanical properties similar to those of blood vessels. Although, this polymer is chemically hydrophobic and is therefore unable to facilitate cell adhesion. It is clear that common surface mobilization techniques such as plasma treatments and then gelatin coating, subsequently trigger platelet aggregation. In this way, there is an urgent need to use anticoagulant drugs such as aspirin and heparin in vascular grafts. If these factors can induce NO production by endothelial cells, there is more hope of solving the problems associated with vascular tissue engineering. In the present study, the optimized concentrations of both compounds were obtained for this application. Specific evaluation methods confirmed 0.05 and 100 mg/mL for aspirin and heparin, respectively. These concentrations related to the half-cell viability (IC_50_) for HUVEC cell line. Although heparin was more potent than aspirin in NO synthesis, these drugs doubled the production of NO due to their synergistic relationship. On the contrary, the increased ability of heparin on platelet activity decreased with aspirin in the combination group. To the best of our knowledge, the faster hydrolysis of heparin compared to aspirin could be the corresponding reason for its better effect. Finally, the stability of this surface modification was examined by measuring aspirin and heparin release over time. The resulting curves confirmed zero order kinetics for both factors. However, the first order pattern and a plateau in particular were detected for heparin. This assay illustrated that the surface modification was retained for at least 10 days. Together, the scaffold offers an idea to prepare substrates with better hemocompatibility.

## Acknowledgments

 This work was funded by Shahid Beheshti University of Medical Sciences under identification number of 10870.

## Author Contributions

**Conceptualization: **Nasim Salehi-Nik.

**Data curation: **Simzar Hosseinzadeh.

**Formal Analysis: **Simzar Hosseinzadeh.

**Funding acquisition:** Nasim Salehi-Nik.

**Investigation: **Nasim Salehi-Nik, Mahboubeh Bohlouli, Nazanin Ghasemi.

**Methodology: **Nasim Salehi-Nik.

**Project administration: **Nasim Salehi-Nik.

**Resources: **Forough Sham.

**Software:** Forough Sham.

**Supervision:** Simzar Hosseinzadeh.

**Validation: **Simzar Hosseinzadeh.

**Visualization: **Elnaz Rostami, Lida Shahghasempour.

**Writing – original draft: **Roya Fattahi, Ghader Nuorooz.

**Writing – review & editing: **Simzar Hosseinzadeh.

## Ethical Issues

 This study was approved by the ethical committee of Shahid Beheshti University of Medical Sciences (ethical number: IR.SBMU.RETECH.REC.1396.12).

## Conflict of Interest

 The authors do not declare any known conflicts with the publication of the data in this study.
